# Sexual Knowledge in Post-Myocardial Infarction Patients: A Cross-Sectional Study

**DOI:** 10.7759/cureus.8480

**Published:** 2020-06-06

**Authors:** Rida Farhan, Rabail Yousuf, Syeda Namayah Fatima Hussain, Maaz Khan, Zara Bilal, Maryam Khan, Zulekha Yousuf, Burhan Khatri, Ayesha Siddiqua, Shenel A Khan

**Affiliations:** 1 Medicine, Dow Medical College, Dow University of Health Sciences, Karachi, PAK; 2 Cardiology, Dow Medical College, Dr. Ruth K. M. Pfau Civil Hospital, Karachi, PAK; 3 Medicine, Liaquat National Hospital and Medical College, Karachi, PAK; 4 Cardiology, United Medical and Dental College, Karachi, PAK; 5 Medicine, Bahria University Medical and Dental College, Karachi, PAK; 6 Internal Medicine, Ziauddin University, Karachi, PAK; 7 Medicine, Jinnah Sindh Medical University, Karachi, PAK; 8 Internal Medicine, Dow Medical College, Dow University of Health Sciences, Karachi, PAK

**Keywords:** sexual knowledge, myocardial infarction

## Abstract

Background

Adequate sexual knowledge is a key component of cardiac rehabilitation. Sexual knowledge in post-myocardial infarction (MI) patients is unknown. Thus, we conducted this study to investigate the sexual knowledge of post-myocardial infarction patients and their accessibility to sexual counseling.

Methodology

Between July and September 2018, a cross-sectional survey was carried out in 6six major hospitals in Karachi. The non-probability convenient sampling technique was used to include all patients meeting the inclusion criteria. To reduce biases, face-to-face interviews were conducted by investigators who were trained prior to the start of data collection. Knowledge was assessed using the Swedish version of the "Sex after MI Knowledge Test" questionnaire, where a higher score meant higher knowledge. Data analysis was done using Statistical Package for Social Sciences (SPSS) version 21.0 (IBM Corp., Armonk, NY) The t-test was applied to compare the knowledge score between males and females.

Results

There was a total of 278 MI patients with a mean age of 54 years, of which 60% were men. The "Sex After MI Knowledge Test" scores varied between 37 and 67 (mean score 51 ± 5). None of the participants obtained the maximum possible test score of 75. The most frequently occurring score was 49 (16%). Around half of the participants (48%) incorrectly believed that sex cannot be safely resumed within a few weeks after the heart attack. Limited knowledge was found in questions pertaining to alcohol, viagra, and late evening being the best time to have sex. Medical knowledge was provided to only 27% (n=76) of the participants, of whom 77% (n=58) received it from the hospital staff itself. A significant difference existed in scores obtained by males and females, with males having a higher score and the mean difference in scores being 1.7 (p=0.015).

Conclusion

Lack of sexual knowledge in MI patients due to the inadequacy of healthcare providers and the social stigma surrounding the topic causes marital strain leading to low quality of life.

## Introduction

It has been predicted that coronary heart disease (CHD) will be the cause of 11 million deaths by 2020 [1-2]. Considering these rising numbers, adequate treatment and recovery following a myocardial infarction (MI) have become increasingly important. It is crucial to maintain an acceptable quality of life and well-being following an MI, amongst which resuming one’s sexual life is also a key. Fear of recurrence, exercise intolerance, and drugs causing sexual dysfunction have known to hinder a patient’s sexual life following an MI [3]. Moreover, adequate sexual knowledge is also a key component of cardiac rehabilitation, as episodic physical and sexual activity is believed to be associated with an increased risk of MI [4].

Studies have shown that normal sexual activity causes a peak heart rate of 125 accompanied by a peak systolic blood pressure of 150-160 mmHg [5]. In MI patients, this increased heart rate adds to the workload of the heart and makes them more prone to a second attack. The drugs usually prescribed post-MI have side effects that impair sexual function, particularly in relation to erectile dysfunction in men [5-8]. Given the effect of these numerous post-MI conditions on the quality of life of patients, there is a need for them to understand the amount of physical activity, including sexual activity, could increase the chances of MI recurrence [9]. However, after hospital discharge, it is generally recommended to resume sexual activity unless stress testing reveals any signs of ischemia or arrhythmias.

Although many patients believe that sexual activity may lead to acute cardiac events, many hesitate to discuss their concerns with their physicians due to the sensitivity of the topic [10-11]. In addition, health care professionals are either uncomfortable about providing any sexual knowledge or they lack the relevant information and competence to communicate about the possible sex-related issues that their patient might face following an MI [12]. Therefore, there is also a need for clinicians to impart appropriate knowledge on the subject, to their patients following their hospital stay after an MI. A study suggested that only one-third of patients received any form of counseling after suffering from a cardiac event [13]. Since there is a paucity of data connecting the impact of MI to the sexual life of the patient, most studies in the past have been noted to focus on pharmacological interventions only, rather than also focusing on the sexual well-being of the individual [14]. Considering the lack of data on the subject, especially in Asia, the primary objective of this study was to investigate the sexual knowledge in patients who had suffered an MI and their accessibility to sexual counseling.

## Materials and methods

Design and setting

This study was conducted over a period of 12 weeks from July to September 2018, to assess the sexual knowledge, awareness, and self-care practices among patients who had an MI in Karachi, Pakistan. This study was approved by the institutional review board of the Dow University of Health Sciences. A non-probability convenience sample was recruited from the wards and outpatient departments of six tertiary care hospitals in Karachi, Pakistan. The inclusion criteria comprised adult patients who were diagnosed with MI a minimum of one month ago by a physician at a hospital and were married and in a sexual relationship with their spouse. The exclusion criteria comprised patients above 75 years of age, those who had MI within the last month, and patients suffering from any mental disability or known sexual problems.

Data collection

In this study, the Swedish version of the “Sex After MI Knowledge Test” was used, which was available in the English language and was also translated to Urdu for people who did not understand English [15]. This 25-item instrument has moderate internal consistency and a Cronbach's alpha of 0.61 [15]. It is a self-rating questionnaire that evaluates the patient’s knowledge regarding the resumption of sexual activity after suffering from an MI. A total of 35 questions were present in the questionnaire and consisted of questions regarding their sexual history and whether the participants had received any prior knowledge concerning sexual health and activity after MI. The second part of the questionnaire assessed the patient’s knowledge concerning sexual activity after MI. It consisted of 25 questions, which were divided into three categories: category one was related to symptoms and physiological reactions during and after sexual activity, the importance of rest, the effect of sexual foreplay, masturbation, and oral and anal sex on heart, the correct time to resume sexual activity and its frequency; category two involved questions related to common emotional responses; and category three involved environmental influences such as medications, alcohol, meals, surrounding conditions, and timing and their effect on sexual activity after MI. In this section, the participants were given three choices (true, false, and do not know) for the statements given, upon which they were scored accordingly. Every correct response (have knowledge) received a score of three and every incorrect response as well as a response of ‘do not know’ was given a score of one. Thus, 75 was the maximum possible score generated with the scores ranging between 25 and 75.

Procedures and ethics

The participants were explained the purpose of the study and verbal consent was obtained before proceeding with the questionnaire. To reduce biases, face-to-face interviews were conducted by the investigators, who were trained prior to the start of data collection. Standard medical terms were also defined and explained to the participants as to eliminate ambiguity. Questions were asked privately, away from other patients, so that they may be able to answer comfortably. They were assured that confidentiality would be maintained, considering the sensitivity of the topic in terms of cultural and social contexts. All interviewers used a standard protocol and any incomplete questionnaires were discarded. The questions were read out to the patients and their responses recorded. A total of 350 responses were collected, in excess of the calculated sample size of 278. The calculated sample size was based on a 50% anticipated frequency from ‘The 25-item Sex after MI knowledge test’ study at 95% CI [15].

Statistical methods

The data were entered and analyzed using the Statistical Package for Social Sciences (SPSS) version 21.0 (IBM Corp., Armonk, NY). Frequencies and percentages were enumerated for categorical responses. The level of sexual knowledge was computed as a continuous variable. Differences in knowledge between males and females were measured as differences between the total scores using the t-test. In all cases, a p-value <0.05 was considered significant.

## Results

The demographic characteristics of the participants are shown in Table 1. A total of 278 participants were included in this study, of which 60% were men. The mean age was found to be 54±8 years. Most of the participants had received school-level education (56%), with only 17% having studied past university level. Ninety-three percent of the participants felt it was "always" or "sometimes" important to have sex. Many of them reported the frequency of having sex as monthly (48%) while 29% experienced discomfort during intercourse. Medical knowledge was provided to only 27% (n=76) of the participants, of whom 77% (n=58) received it from the hospital staff itself.

**Table 1:** Demographic characteristics SD: standard deviation; MI: myocardial infarction

Background Characteristics	Participants n ± SD or (%)
Age, mean ± SD, years	54 ± 8
Sex	
Male	166 (60)
Female	112 (40)
Education	
No education	76 (27)
Up to school	155 (56)
University	47 (17)
Years of relationship with partner	
5 to 20 years	43 (15)
20 to 40 years	202 (73)
40 to 50 years	27 (10)
Above 50 years	6 (2)
Importance of having sex	
Always important	134 (48)
Sometimes important	125 (45)
Never important	19 (7)
Frequency of sex in last 2 months	
Monthly	132 (48)
Weekly	76 (27)
Never	70 (25)
Discomfort during intercourse	
Yes	80 (29)
No	198 (71)
Presence of symptoms	
Always	27 (10)
Sometimes	112 (40)
Never	139 (50)
Medical knowledge regarding sexual activity after MI	
Yes	76 (27)
By hospital faculty	58 (77)
Family and friends	9 (12)
Self-awareness	9 (11)
No	202 (73)

The "Sex After MI Knowledge Test" scores ranged from 37 - 67 (mean score 51 ± 5). No participant managed to obtain the maximum possible test score of 75 in the test, with the most frequently occurring score being 49 (16%). Table 2 shows the results of questions related to symptoms and physiological reactions during intercourse. Emotional reactions and other influencing factors (medications and environmental factors) are shown in Table 3 and Table 4, respectively. The highest scores were obtained on the question "If you have chest pain during sex, you should stop and rest," 89% of the people correctly answered "True." The statements "Masturbation and oral sex are more harmful to the heart than sexual intercourse" and "Anal intercourse can be resumed just as vaginal intercourse since it has less effects on the heart" were answered correctly by only 8% and 7% of the participants, respectively.

**Table 2:** Percentage of correct answers regarding symptoms and physiological reactions in the "Sex After MI Knowledge Test" (category one)

Statements	Correct Answer	Participants n (%)
A danger sign to report to the physician is shortness of breath or increased heart rate	True	244 (88)
Increased heart rate, blood pressure and breathing rate are normal responses during sex	True	152 (55)
If you have chest pain during sex, you should stop and rest	True	247 (89)
Not being able to sleep after intercourse or extreme fatigue the day after intercourse is normal	False	142 (51)
It’s helpful to be rested before intercourse	True	217 (78)
Sexual foreplay when you are more relaxed puts less strain on your heart	True	110 (40)
Masturbation and oral sex are more harmful to the heart than sexual intercourse	False	21 (8)
You should report to your physician a feeling of tightness, fullness, or chest pain during sex	True	244 (88)
If you are tense or tired, you should not have intercourse until after a good night’s sleep	True	219 (79)
Palpitations (rapid heart beating) lasting more than 15 minutes after intercourse are normal	False	139 (50)
Sex can generally be safely resumed within a few weeks after the heart attack	True	94 (34)
Anal intercourse can be resumed just as vaginal intercourse since it has less effects on the heart	False	19 (7)

**Table 3:** Percentage of correct answers related to emotional reactions in the "Sex After MI Knowledge Test" (category two)

Statements	Correct Answer	Participants n (%)
A common emotional reaction after heart attack is depression	True	201 (72)
A good way to ease back into sex is to talk with your partner about your feelings about the heart attack while taking a daily walk.	True	159 (57)
It is normal to feel aggressive or helpless if your partner is overprotective of you after a heart attack	True	145 (53)
You should try not to upset your partner with your fears about resuming sex	False	148 (54)
It is important to have sex as often as before your heart attack	False	181 (65)

**Table 4:** Percentage of correct answers related to environmental and other influencing factors in the "Sex After MI Knowledge Test" (category three)

Statements	Correct Answer	Participants n (%)
Drinking alcohol prior to sex will help you relax and improve sex.	False	37 (13)
Some medicines used for high blood pressure, anxiety, or depression can affect sex.	True	134 (48)
If you think a medicine is causing a problem with sex, you should stop it immediately	False	117 (42)
Late evening or the end of the day is the best time to have sex when you are more relaxed.	False	27 (10)
Wait 2-3 hours after a heavy meal before having sex	True	220 (79)
A room temperature that is not too hot or cold is important for sex	True	185 (67)
Such precautionary measures are helpful	True	208 (75)
Do you think taking drugs like Viagra for erection are helpful	True	37 (13)

**Figure 1:**
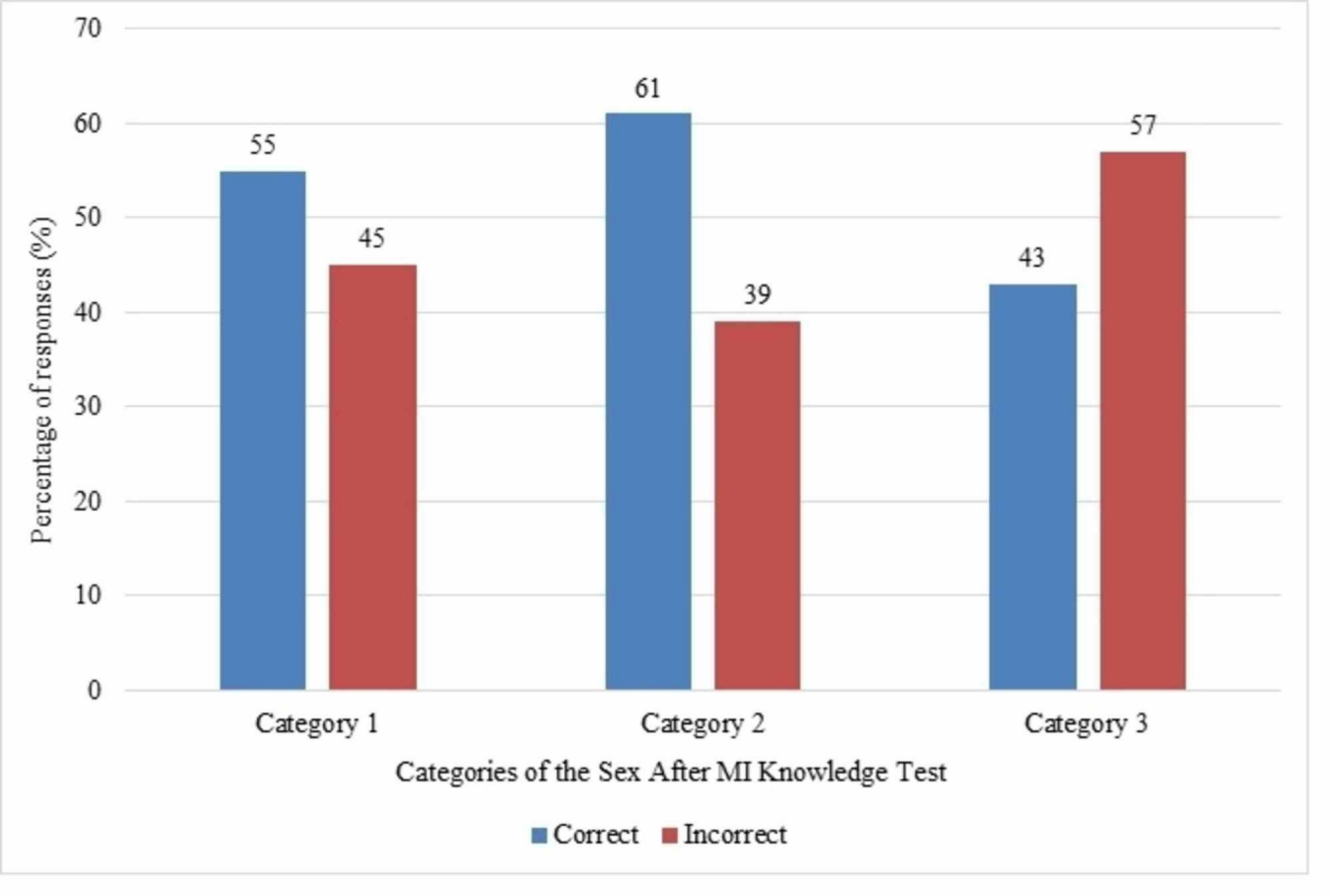
Percentage of correct and incorrect answers in each category of the "Sex After MI Knowledge Test"

**Figure 2:**
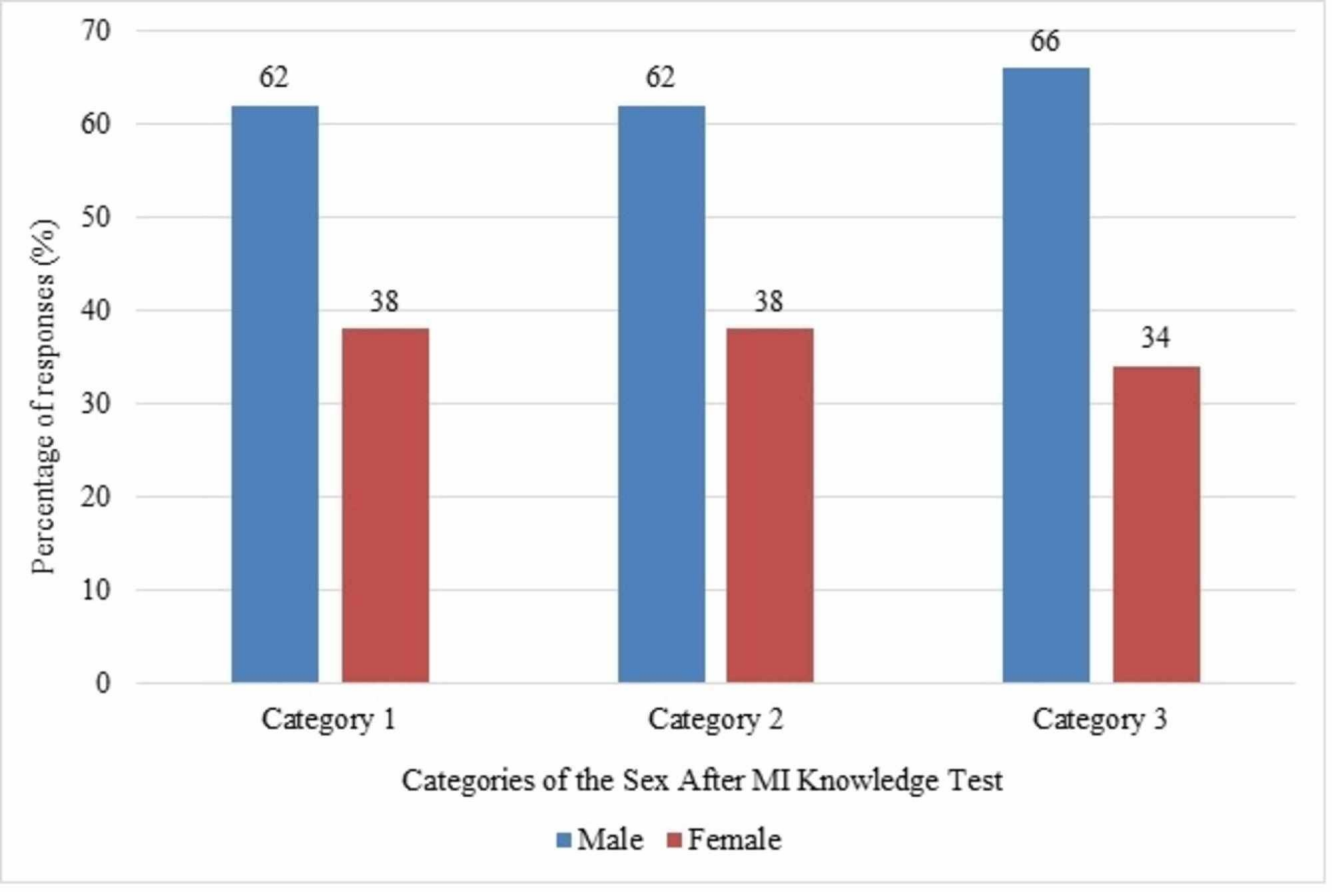
Percentage of correct responses of both genders

Almost half (48%) of the participants incorrectly believed that it was unsafe to resume sex after a few weeks following an MI. Moreover, 18% of them did not know whether this statement was correct or not. Concerning environmental factors, the least amount of knowledge was shown in questions pertaining to alcohol (13%), phosphodiesterase 5 inhibitors such as sildenafil (Viagra) (13%), and late evening being the best time to have sex (10%). Overall, the participants were most knowledgeable in category two (emotional reactions) where 61% of the answers obtained were correct. In category one (symptoms and physiological reactions), 55% of the questions were answered correctly. Less than half of the answers in category three (environmental and other influencing factors) were answered correctly (43%), showing the least amount of knowledge about environmental and other factors such as medications and alcohol use affecting sex (Figure 1). Figure 2 shows the percentage of correct responses by both genders. A significant difference was found in scores obtained by males and females (mean difference in scores was 1.7; p=0.015).

## Discussion

We found that most of the participants had limited knowledge about resuming sexual activity post-MI. Any form of sexual counseling by healthcare professionals was lacking. A previous study conducted showed similar results with a mean score of 51 ± 10, and another study, the Swedish national survey, found that healthcare professionals rarely provided any information regarding sexual activity to post-MI patients or their partners [16-17]. Sexual counseling or openly discussing sexual activity is often considered a shameful topic and is also an old societal taboo, especially in Asian countries like Pakistan. In our study, most participants were noted to be either uneducated or educated up to only the school level, reflective of low socioeconomic status. Therefore, due to the unavailability of resources like the internet or healthcare books, the medical team remains the sole source of guidance regarding any health-related queries they might have. Furthermore, the mean age of the participants with a history of MI was 54 years; an age where the sexual life of most becomes stagnant. Most people of this age, especially those from the lower socioeconomic background, have either already achieved the status of grandparents or are about to become grandparents. Moreover, across the Indian subcontinent, particularly in Pakistan, the joint family system, which consists of many generations living under the same roof, still prevails, which makes undertaking sexual activity for these relatively older individuals an embarrassing task to engage in. Hence, for the reasons mentioned above, most individuals do not feel the need to be sexually counseled or ask for information following an MI.

We found men to have significantly greater knowledge on the subject than women. Several studies have concluded that women were significantly less informed and less likely to receive sexual counseling and that men had comparatively higher knowledge than women regarding the subject [17-19]. This is because, in a patriarchal society, sex is an easily spoken subject among men. It is considered to be a conquest and a matter of pride for them, which is supported by several studies stating that men were generally more curious than women regarding obtaining information about sexual concerns [20-21]. Another reason is that more men suffer from MI (at up to 60 years) as compared to women [22]. Another study showed that since women with coronary artery diseases belonged to a much older age group as compared to men, they were less likely to discuss the matter related to sexual activity with their doctors, who also assume them to be sexually inactive, thus ignoring the need for sexual counseling [23-25]. These findings demonstrate the need for clinicians to provide sufficient information to both the genders equally about resuming sexual activity after MI.

Due to the common knowledge of the physical signs and symptoms of MI, the majority of the participants were aware of chest pain during sex as a danger sign and understood the need to stop and rest. However, owing to the considerable secrecy and mortification associated with sexual intercourse, along with the reluctance of healthcare staff to broach the subject with their patients, almost half of the patients were hesitant to resume sex within the few weeks following an MI. A previous study conducted in Brazil also found that only 4% of the patients received sexual guidance and more than half were doubtful of the acceptability of sexual activity after being discharged from the hospital [26]. This was mostly due to the dread of having re-infarction or even sudden death following any sexual activity [27]. As a consequence, patients are known to suffer from stress, anxiety, and depression, putting a strain on their marital life.

Sexual counseling after an MI is an important factor for recovery, as it helps the patients to ease into their daily routines, avoiding feeling crippled due to an inability to perform well in their sex life [28]. Some other findings from the data collected were limited knowledge pertaining to oral or anal sex and drinking alcohol. This could be because of the majority’s religious beliefs against these topics, prompting an unwillingness to answer such questions. There was also a lack of knowledge about medications and their effects on the heart. The results of this study also disclosed that the majority of the participants chose to readily give up on medication over temporary problems in their sexual activity, hence reflecting upon the personal importance of a healthy sexual life.

Medical knowledge was provided to only 27% (n=76) of the participants, of whom 77% (n=58) received it from the hospital staff. Keeping in view the lack of other sources of information available to these patients, it is essential to train healthcare staff to be better equipped to counsel patients and to encourage them to discuss their doubts more openly. The "Sex After MI Knowledge Test" itself can be an effective tool in not only initiating this discussion but also in giving useful insights as to where the patient lacks awareness. They should be sufficiently reassured that sexual activity is neither a risk factor nor a trigger in inducing another MI, thus alleviating their concerns and facilitating complete recovery [10]. Another way of creating awareness could be the use of informative leaflets and guidelines distributed by clinicians alongside group discussions led by informed healthcare staff [16]. Telephone counseling is also emerging as an effective means of addressing the sexual concerns of the patients while maintaining their anonymity; this, alongside the use of leaflets, could prove to be particularly useful in conservative countries [22].

There are several limitations in our study that should be taken into consideration. First, the data collected were mostly from people of low socioeconomic backgrounds and from government hospitals. Although these are large tertiary care hospitals with people from all over the country being treated there, we believe that including private sector cardiac care institutes would have been a better representative sample. Second, since face-to-face interviews were conducted and a match in sex between the interviewer and participant was not considered, there is a possibility that information was withheld considering the sensitive nature of the topic. Third, since alcohol and anal intercourse are prohibited in the state religion, the results are likely to not be an accurate representation. Fourth, we did not assess the correlation between sexual knowledge and the time duration after MI, nor did we study the sexual knowledge of the partners of MI patients. In the future, detailed studies that include more hospitals and those including the knowledge of both the patient and their partners should be conducted.

## Conclusions

This study was conducted to gain insight into the sexual knowledge of patients who had suffered a myocardial infarction (MI). Owing to the shame associated with anything related to sexual intercourse, MI patients are often deprived of proper counseling after an attack. Given that an inactive sexual life can cause marital problems, disrupting the entire family structure, it is essential for healthcare providers to guide MI patients in terms of sexual activity following their hospital stay. We believe that removing the patient’s fear and doubts about resuming sexual intercourse would help hasten their recovery and alleviate any undue stress caused between them and their partners, hence improving their quality of life.
